# LeishDB: a database of coding gene annotation and non-coding RNAs in *Leishmania braziliensis*

**DOI:** 10.1093/database/bax047

**Published:** 2017-06-13

**Authors:** Felipe Torres, Raúl Arias-Carrasco, José C. Caris-Maldonado, Aldina Barral, Vinicius Maracaja-Coutinho, Artur T. L. De Queiroz

**Affiliations:** 1 Centro de Pesquisas Gonçalo Moniz (CPqGM), Fundação Oswaldo Cruz (FIOCRUZ), Salvador, Brazil; 2 Programa de Pós-Graduação em Computação Aplicada (PGCA), Universidade Estadual de Feira de Santana, Feira de Santana, Brazil; 3 Centro de Genómica y Bioinformática, Facultad de Ciencias, Universidad Mayor, Santiago, Chile; 4 Universidade Federal da Bahia, Salvador, Brazil; 5 Instituto Nacional de Ciência e Tecnologia de Investigação em Imunologia (iii-INCT), São Paulo, Brazil; 6 Beagle Bioinformatics, Santiago, Chile; 7 Instituto Vandique, João Pessoa, Brazil

## Abstract

*Leishmania braziliensis* is the etiological agent of cutaneous leishmaniasis, a disease with high public health importance, affecting 12 million people worldwide. Although its genome sequence was originally published in 2007, the two reference public annotations still presents at least 80% of the genes simply classified as hypothetical or putative proteins. Furthermore, it is notable the absence of non-coding RNA (ncRNA) sequences from *Leishmania* species in public databases. These poorly annotated coding genes and ncRNAs could be important players for the understanding of this protozoan biology, the mechanisms behind host-parasite interactions and disease control. Herein, we performed a new prediction and annotation of *L. braziliensis* protein-coding genes and non-coding RNAs, using recently developed predictive algorithms and updated databases. In summary, we identified 11 491 ORFs, with 5263 (45.80%) of them associated with proteins available in public databases. Moreover, we identified for the first time the repertoire of 11 243 ncRNAs belonging to different classes distributed along the genome. The accuracy of our predictions was verified by transcriptional evidence using RNA-seq, confirming that they are actually generating real transcripts. These data were organized in a public repository named LeishDB (www.leishdb.com), which represents an improvement on the publicly available data related to genomic annotation for *L. braziliensis*. This updated information can be useful for future genomics, transcriptomics and metabolomics studies; being an additional tool for genome annotation pipelines and novel studies associated with the understanding of this protozoan genome complexity, organization, biology, and development of innovative methodologies for disease control and diagnostics.

**Database URL:** www.leishdb.com

## Introduction

Cutaneous leishmaniasis is an important neglected tropical disease that affects mainly human skin and mucosal tissues, causing ulcerated wounds (1, 2). According to the World Health Organization (WHO), the disease infection rate was estimated in 0.7–1.3 million new cases yearly, with about 350 million people under leishmaniasis infection risk worldwide (WHO Leishmaniasis Fact Sheet, March 2016). Currently, 61 countries reported the disease, with only 10 (Afghanistan, Algeria, Brazil, Colombia, Costa Rica, Ethiopia, Iran, Peru, Sudan and Syria) concentrating up to 75% of leishmaniasis cases (WHO Global Health Observatory Data Repository, March 2015). The etiological agent of leishmaniasis are protozoan parasites from the *Leishmania* genus, which are digenetic parasites that develop as promastigotes in the gut of phlebotomine sandflies, and as intracellular amastigotes in the macrophages of vertebrate hosts (3). The genus is composed by several species, including *Leishmania braziliensis*, the most representative in Brazil (4), and responsible for 20 187 new infected people in 2015, according to the Brazilian Unified Health System (SUS) (http://datasus.saude.gov.br).

*Leishmania braziliensis* genome is composed by 34 chromosomes with considerable structural genomic divergences compared to other species, like the fusion between chromosomes 20 and 34 (5). The reference *L. braziliensis* genome available in NCBI database is the strain MHOM/BR/75/M2904, deposited originally by The Sanger Institute (6). There are two main genome annotations available for this strain in public databases. The NCBI (7) annotation presents a total of 8161 predicted coding genes, with almost 86.87% of them (7089) without functional annotation, and classified as hypothetical or putative genes. The other annotation is provided by TriTrypDB database (8), which stores information related to 8505 predicted genes, with 80.51% (6848) of them annotated as hypothetical or putative genes.

The fine-tuning regulation of eukaryotic cells is orchestrated by a myriad of different non-coding RNA classes (9–11), acting as important elements in catalytic and regulatory functions on nuclear and cytoplasmic activities (12). Besides some research groups reported a variety of small and long non-coding RNAs in different *Leishmania* species (13–18), it is notable the lack of information regarding ncRNAs in public databases. It can be evidenced on the number of entries for this molecular type from Leishmania *ssp.* available on public repositories for non-coding transcripts, such as the Non-coding RNA Database Resource (NRDR) (11), which currently integrates data from >150 databases associated with ncRNAs. The current version of NRDR (January 2017) shows the presence of only 324 non-coding RNA sequences from the *Leishmania* genus, and only one of them from *L. braziliensis*, a ribosomal RNA (rRNA).

This study performed a prediction and annotation of *L. braziliensis* protein-coding genes and non-coding RNAs, using recently developed predictive algorithms and updated databases. The reannotation process resulted in the prediction of 11 491 open reading frames (ORFs), with 5263 (45.80%) of them annotated with proteins available in public repositories. Our database represents an increase of at least 26% on the number of coding gene predictions compared to other databases (5, 8). In addition, our annotation process resulted in the identification of 11 243 potential non-coding RNAs from different classes. Both coding and non-coding RNA predictions had their expression validated using RNA-seq public data. To the best of our knowledgement, this is the most comprehensive systematic identification and functional annotation of different regulatory ncRNAs in *L. braziliensis*. All results were organized and deposited on the open-source database named LeishDB, available at: www.leishdb.com.

## Materials and methods

### Datasets and databases

In this study, we used the genome sequence from *L. braziliensis* MHOM/BR/75/M2904, originally published by The Sanger Institute (6). For the protein-coding genes annotation by sequence similarity searches, we used the non-redundant proteins available in NCBI (7) and UniProtKB (19) databases. Gene ontology (GO) terms annotation was performed using the updated Gene Ontology database (20), through AmiGO 2 tool (21). For the non-coding RNAs predictions and annotations, we used the RNA covariance models from all RNA families available in the version 12 of Rfam database (22), and the annotation available in other public repositories retrieved from the Non-coding RNA Databases Resource (NRDR) (11).

### Protein-coding gene predictions and functional annotation

The open reading frame (ORF) identification was performed using five algorithms for protein-coding gene predictions: GENSCAN (23), GLIMMER (24), SNAP (25), RATT (26) and AUGUSTUS (27). GENSCAN parameters were setted to default, while the GLIMMER 3.02 parameters used were: the genomic code setted to 11 and topology setted to ‘linear’. SNAP, RATT and AUGUSTUS predictions were obtained through Companion web server (28), using default parameters. A consensus prediction from all software was generated using BEDTools (29).

FASTA sequences from all coding genes were compared against protein databases described in ‘Datasets and databases’ section using BLASTx algorithm (30). A 50% similarity threshold between elements and an e-value smaller or equal than 10^−5^ was used as cutoff. The functional GO terms identification was performed using AmiGO 2 with standard options (21).

### Non-coding RNA predictions and functional annotation

The non-coding RNAs automatic prediction and annotation were performed using two different approaches, based on covariance models comparisons and sequence similarity searches. Firstly, we impemented the in-house developed pipeline StructRNAFinder (http://integrativebioinformatics.me/structrnafinder/ and Supplementary Figure S1). This tool automatically integrates different widely used tools for ncRNAs prediction and secondary structure identification, such as Infernal (31) and RNAfold (32); with the information available on the RNA families database (Rfam) for functional annotation (32). Infernal was used on the comparisons of all sequences and secondary structures covariance models available in Rfam database, against the *L. braziliensis* genome, using a cmsearch e-value cutoff of 0.001 and score of 10.

The sequence similarity search approach was implemented by comparing all ∼8 million non-coding RNA sequences integrated on the Non-coding RNA Databases Resource (11), against *L. braziliensis* genome sequence using Bowtie2 (33). Mapping redundancies were eliminated using BEDTools (29). RNA classes annotation and original species information were recovered from each sequence used on the mapping.

MicroRNA target predictions were performed through IntaRNA tool (34), using all predicted microRNAs and protein-coding genes as input. A minimum energy of −13.34 kcal/mol was used as cutoff for all microRNA-protein coding genes interactions, as suggested by Lai and Meyer (35).

### Transcriptional evidence for coding genes and non-coding RNAs predictions

In order to obtain additional validation regarding the coding genes and ncRNAs predictions, we performed an expression analysis using the unique RNA-seq dataset (accession number: SRR2767683) available for *L. braziliensis* MHOM/BR/75/M2904 on NCBI SRA database (36). Low quality raw reads were filtered using Trimmomatic, version 0.36 (37), with a Phred score cutoff of Q = 28. High quality reads were mapped against the reference genome using TopHat, version 2.1.1 (38). The expression values for each coding gene or ncRNA were estimated in reads count using HTSeq-count, version 0.7.2 (39).

### Database and web interface implementation

LeishDB database entity-relationship model was built using MySQL WorkBench (www.mysql.com). The database SQL code was exported and manually edited. The final infrastructure was composed by an Apache HTTP Server with PHP 5.7 and MySQL Server 5.5. The web interface was designed using JavaScript, jQuery, CodeIgniter 3.1.2 and Bootstrap Framework CSS. All LeishDB source code is freely available on GitHub platform at https://github.com/fgtorres/LeishDB or https://github.com/viniciusmaracaja/LeishDB, under Creative Commons and Open Source GNU licenses.

## Results

### Updating the prediction and annotation of *L. braziliensis* protein-coding genes

To update the protein-coding gene predictions and annotations of *L. braziliensis* MHOM/BR/75/M2904 genome, we performed a combined ORF prediction approach using five different predictors (GENSCAN, GLIMMER, SNAP, RATT and AUGUSTUS). We chose to use the consensus between all these tools due to the complexity of *L. braziliensis* genome, that besides being an eukaryotic organism, it possess a gene structure composed by polycistronic transcription (3). The intention of using all these approaches was to evaluate all potential gene structure variations occurring in *L. braziliensis*, due to its unusual eukaryotic genomic organization. Together, these tools predicted 11 491 ORFs, with an average length of 964.61 nt and a GC content estimated in 57.72%. In comparison with the current annotation available for this species, with 8505 (TryTripDB) and 8161 (NCBI) predicted protein-coding genes (5, 8), LeishDB represents an increase of at least 26% in the number of predicted genes compared to current available predictions for this species.

**Figure 1.**
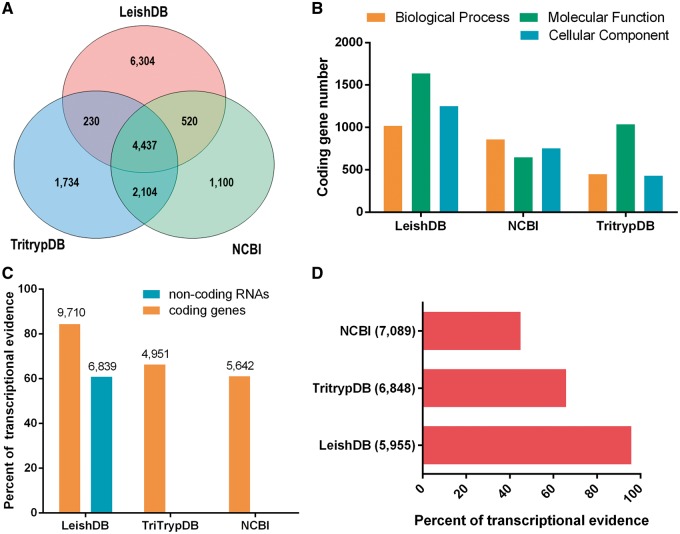
(**A**) Venn diagram comparing *Leishmania braziliensis* MHOM/BR/75/M2904 predicted coding genes available in LeishDB, NCBI and TriTrypDB. (**B**) Gene Ontology (GO) terms comparison between the annotations available in LeishDB, NCBI and TriTrypDB. (**C**) Transcriptional evidence for all predicted coding genes and non-coding RNAs available in LeishDB, NCBI and TriTrypDB. (**D**) Transcriptional evidence for all hypothetical coding genes available in LeishDB, NCBI and TriTrypDB.

Our annotation process identified 48.93% (5623 out of 11 491) predictions associated with proteins available in public databases (including hypothetical proteins). Our gene predictions covered 60.74% (4957 out of 8161) of the predictions available in NCBI and 79.61% (6771 out of 8505) available in TriTrypDB, with an increasing of other 6304 predictions not previously identified (Figure 1A). Considering only predictions annotated with an associated function (excluding hypothetical proteins), we identified a total of 5254 coding genes, which represents 93.43% (5254 out of 5623) of annotated predictions. This is a considerable increase on the number of gene predictions with an associated function compared to NCBI and TritrypDB, which has 2662 and 3472, respectively. The gene ontology annotation process using AmiGO 2 (21), identified a total of 1018 coding genes associated with Biological Process; 1637 with Molecular Function; and 1251 with Cellular Component. Figure 1B demonstrate our GO terms annotations in comparison with NCBI and TriTrypDB databases.

Our pipeline generated a total of 6304 protein-coding gene predictions reported here for the first time. This high number of novel predictions led us to perform a validation using the unique publicly available RNA-seq dataset for this *L. braziliensis* strain. This analysis confirmed that 84.52% (9713 out of 11 491) of all LeishDB predicted coding genes have at least one RNA-seq read count, suggesting a transcriptional evidence for these predictions (Figure 1C). Considering gene predictions without any match with proteins from public databases, we found transcriptional evidence (at least one read count) for 95.60% (5954 out of 6228) of them, giving evidence for its existence. When comparing the transcriptional evidence of gene predictions with an annotated function, 70.89% (3731 out of 5263) presented transcriptional evidence. This number for TriTrypDB predictions was 66.33% (2303 out of 3472), and for NCBI predictions was 61.02% (1624 out of 2661). When considering predictions defined as hypothetical proteins, 95.80% (5955 out of 6216) of LeishDB predictions presented transcriptional evidence. This number for TriTrypDB was 65.82% (4504 out of 6842), and for NCBI was 44.92% (3185 out of 7089) (Figure 1D). We defined as hypothetical proteins all predictions without matching with proteins from public databases, or those containing a match with a protein annotated as hypothetical. The transcriptional evidence was defined by the existence of at least one read count mapping a predicted gene. The number of read counts per gene prediction was recovered and incorporated to the database.

### Non-coding RNAs in *L. braziliensis*: prediction, functional annotation, transcriptional evidence and conservation

Non-coding RNAs predictions and annotations through covariance models comparisons identified a total of 735 ncRNAs in *L. braziliensis* MHOM/BR/75/M2904 genome. Based on Rfam annotation and nomenclature (22), these RNAs were distributed into the following RNA classes: 421 miRNAs, 147 snoRNAs, 2 snRNAs, 11 rRNAs, 76 tRNAs, 10 IRES, 16 sRNAs, 14 lncRNAs and 6 from other classes (Table 1). According to our RNA covariance models comparisons, *L. braziliensis* ncRNAs have a length varying from 34 to 459 nucleotides, and a GC content varying from 50.78% to 60.61% (Table 1). The long length observed for microRNAs is because the prediction was performed considering the nucleotides available on the whole loop responsible for its secondary structure. The small length observed for some long ncRNAs, with sequences smaller than 200 nt, is because the prediction identified secondary structure motifs from long ncRNAs distributed along the genome sequence. The exact length of these lncRNAs should be further confirmed experimentally.

**Table 1.** General overview of all non-coding RNA classes identified in *Leishmania braziliensis* MHOM/BR/75/M2904 genome^a^

Gene type	# of predictions by similarity (%)	# of predictions by covariance models (%)	%GC (SD)	Average length (SD)	Prediction mean by chromosomes
miRNAs	1275 (12.13%)	421 (57.27%)	55.55% (±10.26)	38.53 (±39.52)	48.45
snoRNAs	333 (3.16%)	147 (20%)	53.68% (±13.60)	38.43 (±36.64)	13.71
snRNAs	4 (0.03%)	2 (0.27%)	47.47% (±3.9)	109.25 (±29.19)	0.17
rRNAs		11 (1.50%)	51.43% (±3.33)	117.54 (±2.14)	0.31
tRNAs	479 (4.56%)	76 (10.34%)	65.87% (±12.65)	30.40 (±24.52)	15.85
IRES		10 (1.37%)	60.61% (±5.18)	113 (±45.72)	0.28
sRNAs	1335 (12.70%)	16 (2.18%)	61.37% (±19.95)	21.97 (±18.17)	38.60
piRNAs	598 (5.69%)		63.11% (±14.70)	26.78 (±17.81)	17.08
siRNAs	627 (5.97%)		55.14% (±11.06)	19.53 (±3.14)	17.91
lncRNAs		14 (1.90%)	59.03% (±6.16)	124.78 (±38.32)	0.40
Other or Multiple classes	5857 (55.73%)	38 (5.17%)	53.94% (±16.53)	35.25 (±25.37)	168.42
Total ncRNAs	10 508 (93.46%)	735 (6.53%)	56.14% (±16.22)	26.78 (±28.45)	321.22

a The GC content, average length and the average distribution along the chromosomes are represented. SD = standard deviation.

**Figure 2.**
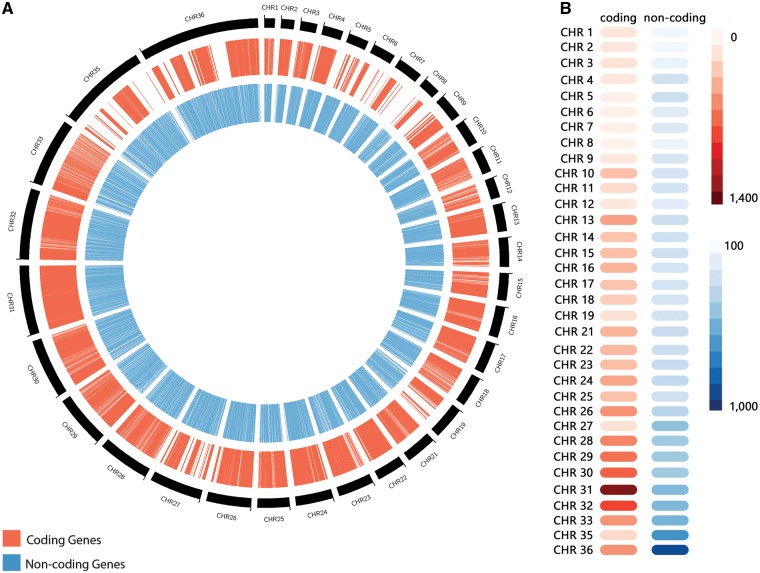
(**A**) Distribution of predicted coding genes (in red) and non-coding RNAs (in blue) along *Leishmania braziliensis* MHOM/BR/75/M2904 chromosomes. (**B**) Heatmap representation of the number of coding genes and non-coding RNAs by chromosome.

The non-coding RNA predictions through sequence similarity search revealed a total of 10 508 RNAs (Table 1), distributed through the following different classes: 1275 miRNAs, 333 snoRNAs, 4 snRNAs, 479 tRNAs, 1335 sRNAs, 598 piRNAs, 627 siRNAs and 5857 from other classes. Those RNAs presented a length varying from 16 to 606 nucleotides, and a GC content varying from 47.47% to 65.87% (Table 1). Altogether, our predictions revealed a total of 11 243 ncRNAs, encompassing a myriad of RNA classes. *L. braziliensis* ncRNAs are distributed along all chromosomes, but an over-representation on regions characterized by protein-coding genes absence was observed, such as the chromosomes 35 and 36 (Figure 2). Chromosome 36 is represented here as the fusion event involving chromosomes 20 and 34, previously reported on literature (6). To the best of our knowledge, this is the first genome-wide systematic identification of non-coding RNAs in *L. braziliensis.*

Our RNA-seq expression analysis for the predicted non-coding RNAs revealed that 60.82% (6838 out of 11 243) presented transcriptional evidence. This lower percentage in comparison to protein-coding genes might be associated with the fact that the RNA-seq library used in this study was developed focused on polyadenylated transcripts, which clearly does not encompass all different ncRNAs that may be non-polyadenylated (40, 41). Additionally, in eukaryotic organisms non-coding transcripts are known by its stage, tissue and cell-specific transcriptional patterns (9, 42, 43) compared to protein-coding genes.

In order to gain further insights related to the conservation of predicted non-coding RNAs, we retrieved the species associated with each one of the ncRNAs identified through sequence similarity search. This analysis revealed a high number of RNAs conserved with higher eukaryotes, specially model organisms (*Homo sapiens*, *Rattus norvegicus*, *Mus musculus* and *Drosophila melanogaster*). The top 10 conserved organisms are listed on Table 2. Nine different Trypanosomatid species presented conservation with *L. braziliensis* ncRNAs, listed on Table 3. This small number of conserved ncRNAs with closely related organisms is clearly result of the absence of data for protozoan parasites in public databases. The list of conserved species for each one of the ncRNAs obtained from sequence similarity searches were recovered and stored in LeishDB.

**Table 2.** Top 10 species presenting conserved non-coding RNAs with *Leishmania braziliensis* MHOM/BR/75/M2904, according to the information retrieved from NRDR database (11)^a^

Species	# of conserved ncRNAs (%)	%GC (SD)	Average length (SD)	# of ncRNA classes
*Homo sapiens*	5502 (52.36%)	54.82% (±16.63)	27.89 (±23.39)	7
*Rattus norvegicus*	4130 (39.30%)	52.71% (±6.76)	30.00 (±15.07)	6
*Mus musculus*	3057 (29.09%)	52.94% (±9.34)	33.99 (±21.17)	6
*Drosophila melanogaster*	2183 (20.77%)	57.45% (±16.92)	21.02 (±11.36)	2
*Chlamydomonas reinhardtii*	1293 (12.30%)	56.05% (±20.90)	28.60 (±39.39)	1
*Arabidopsis thaliana*	255 (2.42%)	54.25% (±14.49)	34.65 (±25.98)	2
*Ozyra sativa*	215 (2.04%)	74.77% (±15.97)	66.33 (±15.19)	1
*Zea mays*	105 (0.99%)	70.75% (±10.02)	26.52 (±14.25)	1
*Leishmania major*	69 (0.65%)	57.60% (±4.01)	74.36 (±4.01)	3
*Trypanosoma brucei*	68 (0.64%)	57.10% (±51.14)	81.79 (±35.85)	3
Other 66 species	293 (2.33%)	53.30% (±11.93)	35.67 (±34.42)	9

a The GC content, average length and the number of conserved ncRNA classes are represented. SD = standard deviation.

**Table 3.** List of all nine *Trypanosomatidae* organisms presenting conserved ncRNAs with *Leishmania braziliensis* MHOM/BR/75/M2904, according to the information retrieved from NRDR database (11)^a^

Species	# of conserved ncRNAs (%)	%GC (SD)	Average length (SD)	# of ncRNA classes
*Leishmania major*	69 (0.65%)	57.60% (±4.01)	74.36 (±4.01)	3
*Trypanosoma brucei*	68 (0.64%)	57.10% (±51.14)	81.79 (±35.85)	3
*Leishmania tarentolae*	35 (0.33%)	56.44% (±5.09)	80.44 (±19.46)	2
*Leishmania mexicana*	4 (0.03%)	56.62% (±4.70)	81.75 (±11.84)	2
*Trypanosoma cruzi*	2 (0.01%)	43.64% (±2.30)	150 (±1)	1
*Leishmania donovani*	2 (0.01%)	54.16% (±5.95)	84 (0)	2
*Leishmania amazonensis*	1 (0.00%)	41.33% (0)	150 (0)	1
*Leishmania enriettii*	1 (0.00%)	41.33% (0)	150 (0)	1
*Phytomonas sp.*	1 (0.00%)	48.51% (0)	101 (0)	1

a The GC content, average length and the number of conserved ncRNA classes are represented. SD = standard deviation.

Our analysis revealed a total of 1696 microRNAs distributed along *L. braziliensis* MHOM/BR/75/M2904 genome. A target prediction search using IntaRNA tool (34), showed that 1666 microRNAs presented 8494 potential target protein-coding genes (minimum energy < −13.34 kcal/mol). The information related to each protein-coding gene predicted as microRNA target was recovered and integrated in LeishDB.

### The generic LeishDB MySQL database model

**Figure 3.**
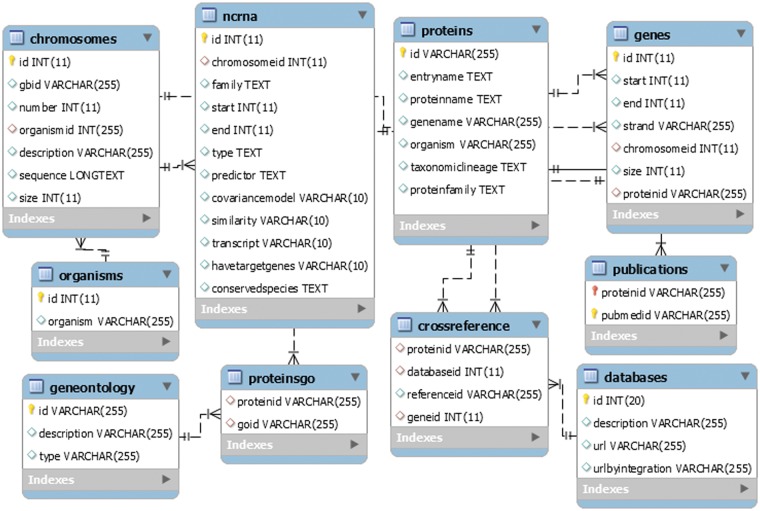
The generic LeishDB entity-relationship model. The DDL (Data Definition Language) and DML (Data Model Language) codes for this database can be downloaded at www.leishdb.com.

LeishDB was created using MySQL as database management system. It was developed as an open source generic model, flexible to be used in other different annotation projects. It can store a myriad of annotation data from multiple organisms, such as chromosomes, genes, ncRNAs, proteins, gene ontology, associated publications, genomic coordinates, sequences, etc. Figure 3 shows a representation of the LeishDB entity-relationship model. The DDL (Data Definition Language) and DML (Data Model Language) scripts can be freely downloaded at: http://leishdb.com/.

### User interface and data retrieving

**Figure 4.**
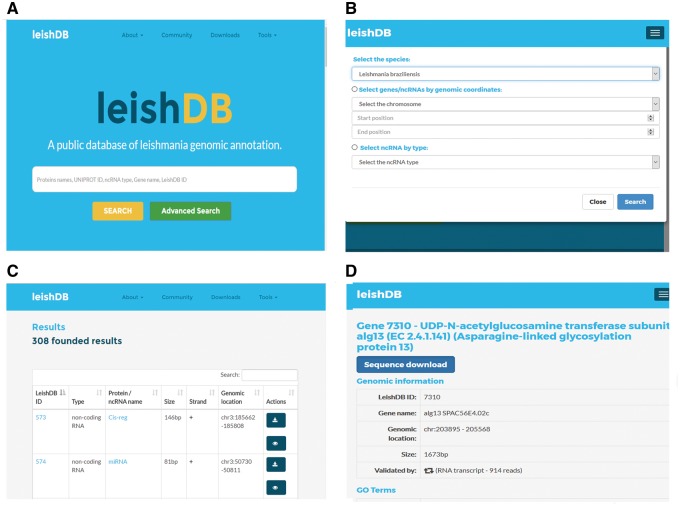
Search methods and results pages from LeishDB. (**A**) Simple search using user defined free-text keywords. (**B**) Advanced search using a chromosome, a genomic coordinate or a ncRNA class of interest. (**C**) Search result containing a list of retrieved predictions according to the search performed. (**D**) Specific page for a particular coding gene or non-coding RNA of interest.

LeishDB provides a search interface where users can retrieve data by different search methods according to user criteria. Free-text searches are available for a simple search where keywords can be applied. As an example, the user can search a *L. braziliensis* coding gene or non-coding RNA by using protein name, UniProt ID, ncRNA class, gene name or LeishDB ID (Figure 4A). Additionally, the user can perform an advanced search by selecting all predictions and annotations available in a particular chromosome of interest; by a particular RNA class of interest; or those available in a particular genomic region of interest, by using genomic coordinates (Figure 4B). The search results present a list of the annotations available containing the keyword, chromosome, RNA class or genomic coordinates of interest retrieved from our server (Figure 4C). By clicking on a retrieved coding gene or ncRNA of interest, the user is redirected to the annotation page itself for that particular gene/ncRNA. This page contains all information indexed in LeishDB after our prediction and annotation process for both coding genes and ncRNAs (Figure 4D). Cross-referencing information is also available for connecting the prediction with several other databases, such as Gene Ontology (20), UniProt (19), EMBL (44), The Protein Model Portal (45), BioGRID (46), STRING (47), PRIDE (48), KEGG (49), Ensembl (50), eggNOG (51), InParanoid (52), InterPro (53), TryTripDB (8) and NRDR (11).

Sequence similarity search was made available through the implementation of BLAST suite by Sequence Server (54), and can be accessed by clicking on the link available on the menu tab ‘Tools’. The genome browser JBrowse (55) was implemented and can be accessed through the annotation page from a particular coding gene or non-coding RNA, or directly by clicking on the link available on the menu tab ‘Tools’. This browser is useful for a general overview of the genomic structure and organization of *L. braziliensis*. Since some of our predictions might not be equivalent with those from TriTrypDB and NCBI databases, we included tracks for both databases in LeishDB genome browser.

LeishDB also provides a digital forum for community participation, which can be accessed by clicking on the link ‘Community’. This resource was developed to strengthen the relationship with final users. Our team will provide continuously updated information through this channel, which will also be used for online discussions between *Leishmania* research community, as well as for receiving feedback, suggestions and criticisms related to our database. For instance, by this communication channel, users will be able to suggest the inclusion of novel genome sequences and annotations, which will be made available after proper review of the database curators.

## Discussion

In this work, we performed an extensive genome-wide prediction of protein-coding genes and non-coding RNAs in *L. braziliensis* MHOM/BR/75/M2904, using updated databases and predictive tools. Generated data were organized and stored in a novel public repository named LeishDB. Firstly, we predicted the repertory of protein-coding genes spreaded over *L. braziliensis* genome using a combination of five different algorithms (GENSCAN, GLIMMER, AUGUSTUS, SNAP and RATT). Our predictions resulted in 11 491 ORFs, with 54.86% (6304) of them predicted for the first time based on a comparison with the predictions available in TriTrypDB and NCBI databases. RNA sequencing public data supported the existence of LeishDB predictions, with 84.50% of them (9710 out of 11 491) presenting transcriptional evidence based on read counts. In summary, 45.80% (5263 out of 11 491) of LeishDB predictions presented match with proteins available in public databases, with 45.72% of them (5254 out of 11 491) presenting functional annotation (e.g. excluding hypothetical proteins). This number of functionally annotated coding genes is a considerable improvement compared to NCBI and TriTrypDB databases, which contains 13.13% (1072 out of 8161) and 19.48% (1657 out of 8505), respectively, of functionally annotated predictions.

Additionally, we performed a genome-wide prediction of non-coding RNAs based on covariance models comparisons and sequence similarity searches approaches. Our predictions revealed the potential presence of 11 243 non-coding RNAs from different classes spreaded over *L. braziliensis* genome. This is the most comprehensive non-coding RNAs prediction and annotation effort for *Leishmania* species. The accuracy of our ncRNA predictions was verified by estimating their transcriptional evidence using publicly available RNA-seq dataset, suggesting that they are actually generating real transcripts. We found transcriptional evidence for 60.82% of the predicted non-coding RNAs. It is important to mention that in eukaryotic organisms non-coding transcripts are known by its stage, tissue and cell-specific transcriptional patterns (9, 42, 43), compared to protein-coding genes. This information may suggest that the remaining 39.18% of non-expressed ncRNAs could have their existence confirmed with the continuous generation of novel RNA-seq datasets. A conservation analysis revealed that the set of predicted ncRNAs was conserved with 76 different species, at different levels of conservation. In general, most of predicted ncRNAs were conserved with model organisms, with only nine species belonging to Trypanosomatidae. This is expected due to the absence of data from these organisms in ncRNA public databases (11).

All the information generated was stored in a public repository for *Leishmania* genomic information. The data can be searched and retrieved using five different search methods, according to user defined criteria: (i) by text-free keyword simple search; (ii) by using the genomic coordinates of a particular region of interest; (iii) by selecting the RNA class of interest; (iv) by navigating on the genome through the available genome browser; or (v) by sequence similarity searches using the integrated BLAST tool. LeishDB source code is freely available and can be used in any genome annotation project.

LeishDB represents an improvement on the publicly available data related to genomic annotation for *L. braziliensis*. This updated information is crucial for the understanding of this protozoan genome complexity, organization, biology, and the mechanisms behind host-parasite interactions. In particular, it can be useful for future transcriptomics, genomics and metabolomics studies; being an additional tool for genome annotation pipelines and novel studies associated with the development of innovative methodologies for the disease control and diagnostics. Our team is currently working on the prediction and annotation of other *Leishmania* species, which will be gradually inserted into LeishDB, in order consolidate this database as a genomic reference repository specific for *Leishmania* spp.

## Supplementary Material

Supplementary Data
