# Supplementary figures and images for: LeishDB: a database of coding gene annotation and non-coding RNAs in Leishmania braziliensis
Source: Database (Oxford). 2017 Jun 13;2017:bax047. doi: 10.1093/database/bax047 (PMC5502370; doi:10.1093/database/bax047)

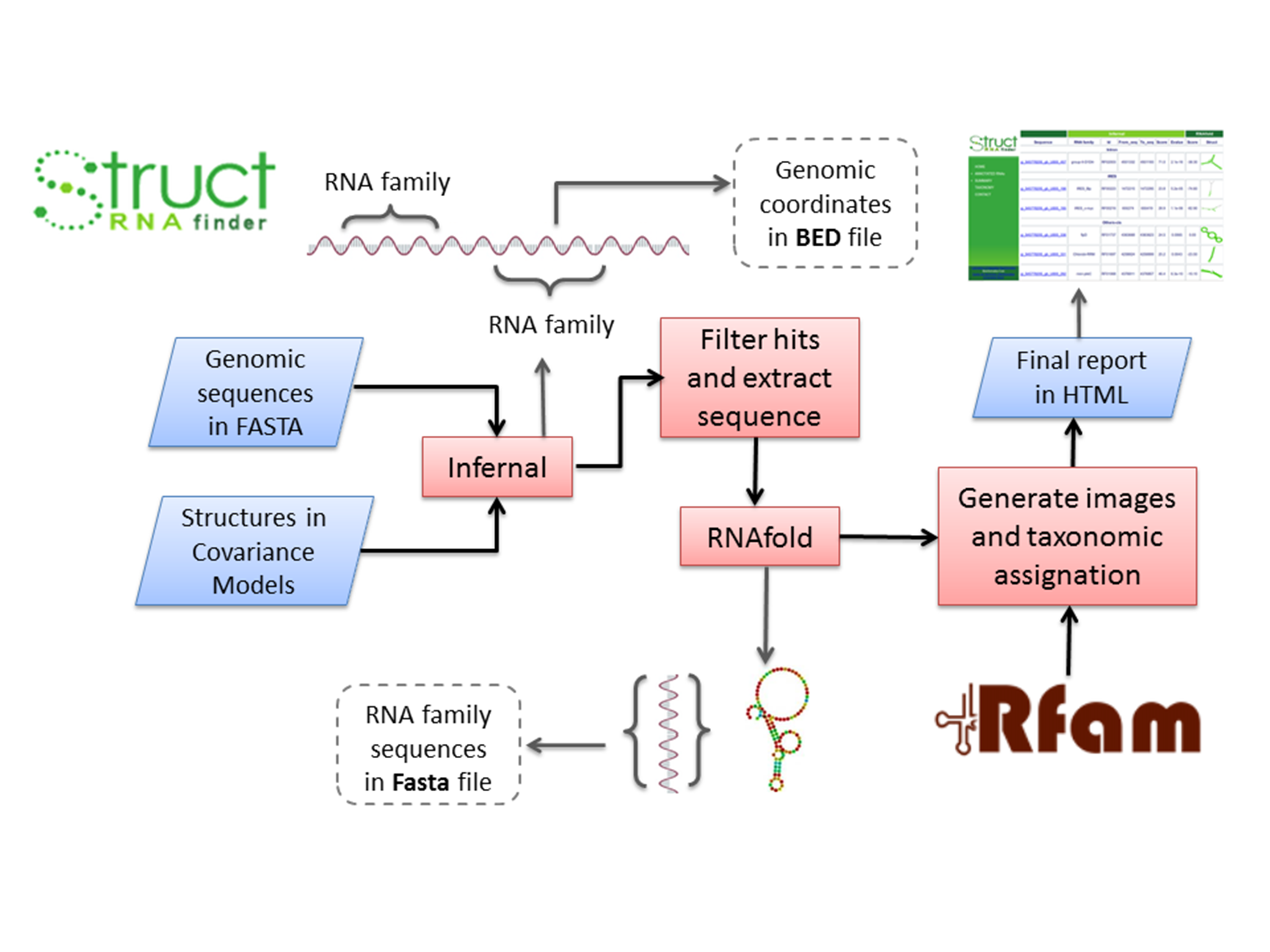

Supplement: Supplementary Data [file bax047_Supp.png]
